# A Flute, Musical Bows and Bamboo Clarinets that “Speak” in the Amazon Rainforest; Speech and Music in the Gavião Language of Rondônia

**DOI:** 10.3389/fpsyg.2021.674289

**Published:** 2021-12-21

**Authors:** Julien Meyer, Denny Moore

**Affiliations:** ^1^Université Grenoble Alpes, CNRS, GIPSA-Lab, Grenoble, France; ^2^Museu Paraense Emílio Goeldi, Ministério de Ciência, Tecnologia e Innovação, Belém, Brazil

**Keywords:** Gavião of Rondônia, speech-music relation, Instrumental speech, talking musical instruments, tone language, archaic speech, musical acoustics, speech surrogates

## Abstract

The Gavião, a native Amazonian group in Rondônia, Brazil, use three different traditional musical instruments that they identify as “speaking” ones and that are characterized by a very tight music-lyric relation through similar pitch patterns: a flute (called *kotiráp*), a pair of mouth bows (*iridináp*), and three large bamboo clarinets (*totoráp*), played by three different players, each one playing a single-note clarinet. They show in different ways the relation of acoustic iconicity which exists between the words of the songs’ lyrics and the music played on such instruments to “sing” the songs. Linguistic analysis makes it possible to understand the phonetic and phonological nature of the iconicity. The sung speech form, being intermediate between the spoken and the instrumental forms, is useful for both learning and explaining the musical notes. In a language with distinctive tone and length, such as Gavião of Rondônia, the first question about speech that is played by musical instruments is the relation between the melodies and the supersegmental phonology of the corresponding words in sung speech and in modal spoken speech. It is influenced by the phonological possibilities of the spoken form and by the musical possibilities of the instrumental form. The description and analysis of Gavião instrumental speech and song practices are found to be a noteworthy contribution to the typology of instrumental language surrogates associated with a tone language, one that calls for a reexamination of hypotheses about which aspects of the phonological/phonetic structure can be transposed in instrumental speech and how this can be done. The role of this kind of instrumental sung speech is artistic and also practical as it contributes to maintain the oral heritage. Such practice represents a little-studied and threatened cultural heritage of the traditional substratum of the cultures of Amazonia.

## Introduction

### Play, Sing, Speak, Sound Structures in Common

Playing musical instruments and singing are considered as independent activities in some cultures/traditions. In fact, songs can exist which are not accompanied by played music and played music can exist without lyrics. However, there are various possible relations between the two. For example, in many musical styles of the world, there is music which has an associated song, even if the song and the music are not always produced together. In various cultures, there is such a strong link between played music and its associated lyrics that they share several sound structures. Among the Gavião of Rondônia (“gavião” means “hawk” in Portuguese, which is a direct translation of the self-denomination “*ik*
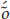
*ló*”—the plural “*ik*
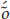
*léèy*” is often preferred by the community) living in the western Amazon in Brazil, a subset of the musical traditions is based on such a strong link. There exist three types of instrumental music—corresponding to three different types of musical instruments: a pair of musical mouth bows, a flute and an ensemble of three one-note bamboo clarinets—in which the instrumental melodies are based on the structure of the language via the emulation of some aspects of the lyrics. The perception of the Gavião is that the instruments are speaking, or, more exactly, that they are expressing the sung form of speech. From this comes the idea of “singing” instruments. The songs are taught to facilitate learning the instrumental music, but the players rarely use them on other occasions. Hearing the melodies played, non-Gavião-speaking observers don’t suspect the relation that they have with the associated lyrics.

### Singing Instruments in the Amazon and in the World

Present in various parts of the world, the phenomenon of instruments which “sing” (imitate aspects of the phonetics and phonology of the words associated with the music) represents a verbal art of traditional musical instruments. This practice has been described for a very large diversity of instruments, ranging from percussion instruments (such as bells, drums, gongs and traditional xylophones; e.g., Sebeok and Umiker-Sebeok, 1976; Zemp and Soro, 2004; McPherson, 2018) to wind instruments [such as flutes (e.g., Ritzenthaler and Peterson, 1954; Hill, 1993; Mindlin et al., 2001), mouth organs (e.g., Catlin, 1982), bamboo clarinets (e.g., Moore and Meyer, 2014), etc.] and to several types of string instruments [such as mouth bows (e.g., Arom, 1970; Yegnan-Touré, 2008, Moore and Meyer, 2014), or even traditional guitars (e.g., Wedekind, 1983)]. Such instrumental emulation of sung speech has been little studied in Latin America, though the Amazonian basin is one of the rare areas with a great diversity of indigenous languages still expressed in this manner. Some publications indicate that this particular practice was observed in the past in the region (Whiffen, 1915; Izikovitz, 1935), while other more recent references show that it still exists in different forms at least in the cultures of the Bora (Thiesen, 1969; Meyer et al., 2012), Cinta Larga (Ermel, 2004), Gavião (Mindlin et al., 2001; Meyer and Moore, 2013; Moore and Meyer, 2014), Kuikuro (Franchetto and Montagnani, 2011), Pirahã (Everett, 1983), Wakuenai (Hill, 1993), and Aikanã (van der Voort, 2016).

Considering other parts of the world, the singing mode of musical instruments was largely studied on the west coast of Africa and is recognized as one of the important characteristics of the musical heritage of the cultures of this region (Carrington, 1949; Nketia, 1976; Sebeok and Umiker-Sebeok, 1976; Locke and Agbeli, 1981; Zemp and Soro, 2004; Kawada, 2014; Winter, 2014; McPherson, 2018; Dentel and Meyer, 2020). This linguistic-musical phenomenon has also been indicated as very common in the southeast of Asia (Stern, 1957; Catlin, 1982; Poss, 2005; Meyer, 2007), and in Papua New Guinea (Niles, 2010), with various types of instruments in each place. The notion of a *singing mode* of the musical instruments was clearly defined by Nketia (1976) to distinguish, in the Akan culture of Africa, the beats of drums used for telecommunication (*speaking mode* imitating spoken speech), from the beats tied to the songs (*singing mode* imitating sung speech). This distinction between speaking mode and singing mode of the drums also exists in Amazonia, and it has been mentioned for example in the culture of the Bora (Thiesen, 1969; Meyer et al., 2012; Seifart et al., 2018). For the Gavião of Rondônia, an equivalent distinction exists between whistled speech, which serves to imitate spoken speech in the case of long-distance communication, and instrumental speech, which imitates songs presented in this study (and see also Meyer and Moore, 2013; Moore and Meyer, 2014; Meyer, 2015).

### Context and Objectives of the Present Study

Although the works cited above represent an important advance, they are still few, and many questions remain without answers about the linguistic functioning of these speech systems. In the case of the Amazonian languages, there are hardly any systematic studies in scientific linguistics about this subject. This fact alone is sufficient to justify the development of an initiative to document sung instrumental speech. Also, an important fact is that, in general, instrumental forms of speech are lost before the forms of speech which only involve the voice. In the case of the Gavião of Rondônia, this situation is worrisome, since it is symptomatic of the threat which weighs on all the oral heritage.

Given the reduced degree of transmission between generations, the Gavião who care about the maintenance of traditional culture think that it is important to have scientific accompaniment for preservation. The capacity to play music which imitates the sounds of its associated song requires a complex understanding, involving the control of various different techniques, for example, the manufacture of the instruments, the learning of the vast repertoire of songs or the playing of musical notes corresponding to words. Sometimes it also requires coordination between different people involved in the music or the simultaneous performance of dances by the players in the context of a festival. Aside from this, the texts regularly make reference to the traditional cosmology of the Gavião and to the relation between the people and the Amazonian environment; participating in its living day-to-day activities. Because of this, these cultural practices suffer invasive and destructive pressure from religions that have been recently imported, who denigrate them and, at times, prohibit them. By substituting texts of traditional beliefs by other texts, the non-native religions eliminate traditional forms of indigenous arts, aside from the traditional mythical vision of the natural and supernatural world of the people.

As part of the response to this urgency, both scientific and cultural, the present article provides a general view of the process of documenting three principal musical instruments identified as “singing” by the Gavião collaborators and describes the relation of acoustic similarity which exists between the musical melodies and the corresponding words in their associated songs, or even in modal spoken speech. Linguistic analysis is important for understanding the phonetic and phonological nature of this acoustic iconic relation, which is one of the bases of the creativity of the musical memory of the Gavião people. Their instrumental speech and singing show previously undescribed typological properties that have scientific importance.

## Methodology

### Steps of the Research

The multidisciplinary methodology that was adopted registered various complementary aspects of the traditional use of the three instrumental speech forms under study. It was carried out in various villages on the Indigenous Territory Igarapé Lourdes in the state of Rondônia in western Brazil. Each step was observed and recorded in video and/or audio. The steps can be replicated and adapted to other cultures with similar practices.

The first step was to identify the instruments involved by making an ethnographic enquiry with various traditional indigenous collaborators in various villages. After the identification of the instruments and of the few players regarded as skillful representative performers, the second step consisted of documenting the players’ preparation for playing in several villages: gathering the materials in the forest, manufacturing the instruments, and tuning the notes that they produce.

In the third step, documentation was carried out of each musical piece and its associated song in various ways, each focusing on a different aspect. First, the instrumental music was played, recorded, and described in its natural context, or, at least in a simulation of the natural context. Second, the associated song, without the instrumental music, was sung, recorded, and transcribed. The content of the lyrics was described ethnographically. Fourth, the lyrics of the songs were recorded in their modal spoken form (not sung). They were then transcribed and glossed as texts using methods which presuppose a reasonably complete description of the language as well as the help of an indigenous consultant to pronounce and translate the words, to respond to analytic questions, and to help identify some aspects of the language used for singing, which is different from modal spoken use. One problem in this step is the difficulty in suppressing the impulse of the informants to sing the lyrics instead of speaking them. Another interesting difficulty is identifying and understanding the archaic or rare forms in the lyrics that are quite common in old songs. Lastly, the three melodies were compared, that of the music played by instruments, that of the associated song, and that formed by the tone and length of the associated song lyrics as they are normally spoken.

Three other steps were: (1) storing the data in two professional linguistic archives [one international (Meyer, 2014) and one national (Moore and Meyer, 2015)], (2) editing video documents for the Gavião villages to return the data to the community in a ready-to-use form (edited documents on CDs, DVDs, pen drives, printed forms that can be used in families, schools, associations), and (3) making the results of the study primarily accessible both locally to the Gavião community—in accordance with the fieldwork authorizations delivered (see next section “Ethics and Permits”)—and nationally to the Brazilian community by publishing them in Portuguese (except young Gavião children and very old people, all speak some Portuguese) (Meyer and Moore, 2013). These steps were important because these singing Gavião musical instruments represent a sociocultural patrimony threatened by extinction. It has equivalents in other Amazonian cultures that are rarely documented due to the fact that they are only maintained in rather isolated cultural communities. Another objective was therefore to stimulate the investigation of instrumental speech by Brazilian linguists and anthropologists. Finally, concerning the Gavião community, the collaborative work was well accepted, especially among the players, who saw their traditional knowledge gaining prestige; among the young people, who recorded sounds and videos and followed the process of edition of DVDs; and among the children, who learned the ancient songs and followed the manufacturing of the traditional instruments. It was also an opportunity to interact with indigenous school teachers about the tone in their language.

### Ethics and Permits

Research was conducted in accordance with the Declaration of Helsinki. In Brazil, there was at that time no standing ethics committee at the Museu Goeldi, our host institution in Belém. Ethical questions, should they arise, were addressed by an internal investigative committee called a “sindicância.” In Brazilian law and practice, the participating indigenous community indicates, either orally or in writing, their informed consent to the proposed research to the local office of the National Indian Foundation (FUNAI), which in turn transmits that consent, in the form of a document, to the central FUNAI office in the national capital. This office issues written research permits. Our research followed these established procedures. Native local authorities authorized our work in all of the visited communities. Permits were obtained from the National Indian Foundation (FUNAI) and the National Research Council (CNPq). Copies of the recordings and the research results were given to the community.

### Some Phonetic and Phonological Characteristics of the Language of the Gavião of Rondônia

It is essential to know some technical aspects of the language of the Gavião to understand the relation between modal spoken speech, the song and the instrumental music associated with each song. Basically, all the Gavião (population of approximately 800 people) speak the language, which is part of the Mondé branch of the Tupi family. In the phonology there are 18 consonant phonemes, not much different from the consonants of English. There are only five contrastive vowels. Nasalization is contrastive on vowels and spreads to the right under certain conditions. There are no stress contrasts (Moore, 1984). The IPA symbols for the consonant phonemes are [*p, t, c, k, ʔ, b, d, 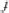, g, m, n, ŋ, ts, dz, β, ɾ, Ɩ, j*]; for the vowels [*i, e, a, o, ɨ*]. In the practical transcription used here, the symbols *c* and *j* denote palatal stops, variably affricated, *y* the palatal glide, and *s* and *z* dental affricates. The voiced bilabial fricative is indicated by *v* and the glottal stop by an apostrophe. The high central vowel is indicated by *u*.

The supersegmental phonology is complicated and quite complex to represent in practical transcription. There are contrasts between high, low and rising tones and also between short and long vowels. Long vowels are phonologically different from two short vowels in the same syllable, though they are phonetically the same. Also, some long syllables have a floating low tone finally. A more detailed analysis of the tonal system is given in Moore (1999) and Moore and Meyer (2014). The transcription used here, originally adapted to the Brazilian keyboard and carried over, for the sake of consistency, from earlier stages of phonological analysis, is adequate for our purposes.

In our transcription low tone is unmarked; high tone is marked by an acute accent and rising tone by a circumflex. Long vowels and sequences of two vowels in the same syllable are both transcribed as sequences of two vowels. In the transcribed examples below all syllables with two equal vowels with low tone are long vowels, not short vowel sequences, for example, *pagátaa* “cut us.” A grave accent is used on the second vowel to indicate long tones which have final floating low tones. The common short and long tones and their phonetic manifestation are given below:


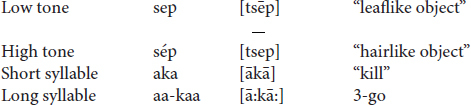


Some long tones can be realized phonetically as contours, for example, the long rising tone or the two long tones which drop as the floating low tone attaches to the end of the syllable when nothing follows:


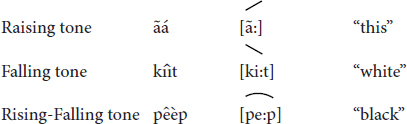


Another complication is that certain syllables (those with an underlying long syllable with a final floating low tone) trigger a fall in the phonetic level of any immediately following high tone to a mid-level. This lowers the register of all following high tones. If nothing follows the syllable, the floating low tone attaches to the end of the syllable and the result is a falling tone, for example: H:(L) > HL. If the syllable is followed by a high tone, the floating low tone attaches to the following high tone and the combination is realized phonetically as a mid-level, for example: H:(L) H > H:^!^ H. So this *downstep*, which is well known in Africa, occurs only in these cases in Gavião and the language does not display downdrift, i.e., H pronounced lower after an overt L tone. Downstep tones, which are systematically rendered by instrumental speech in Gavião (see details in section “Correspondence Between Musical Notes and Phonetic Tone—Playing Flattened Surface Tone”) are quite frequent in Gavião: the final tones of adjectives are generally downstep tones; long final tones in nouns are usually downstep tones, except in monosyllable nature items, where they are level. Long final verb tones are also level, with no floating low tone.

Without going into the details of Gavião tonal phonology, it is worth noting that another phenomenon that is relevant for instrumental singing is low-tone dissimilation, which follows the pattern of the Obligatory Contour Principle (Odden, 1986). Long low tones before immediately following low tones, across word boundaries, optionally (but frequently) become long rising tones: L: L > LH L. This is another postlexical tone alternation which is played by instruments (see details in the Results section “Correspondence Between Musical Notes and Phonetic Tone—Playing Flattened Surface Tone”) as well as spoken and sung.

Speakers of languages without tone and length contrasts generally have considerable difficulty hearing the tone and length. The Gavião, however, easily hear the tone and the length, which have contrastive weight in their language. These phonological traits are the principal base of the whistled speech of the Gavião and also the iconic resemblance between speech, songs and instrumental music (Moore and Meyer, 2014; Meyer, 2015) as we will describe more thoroughly in the present paper.

## Results

**FIGURE 1:**
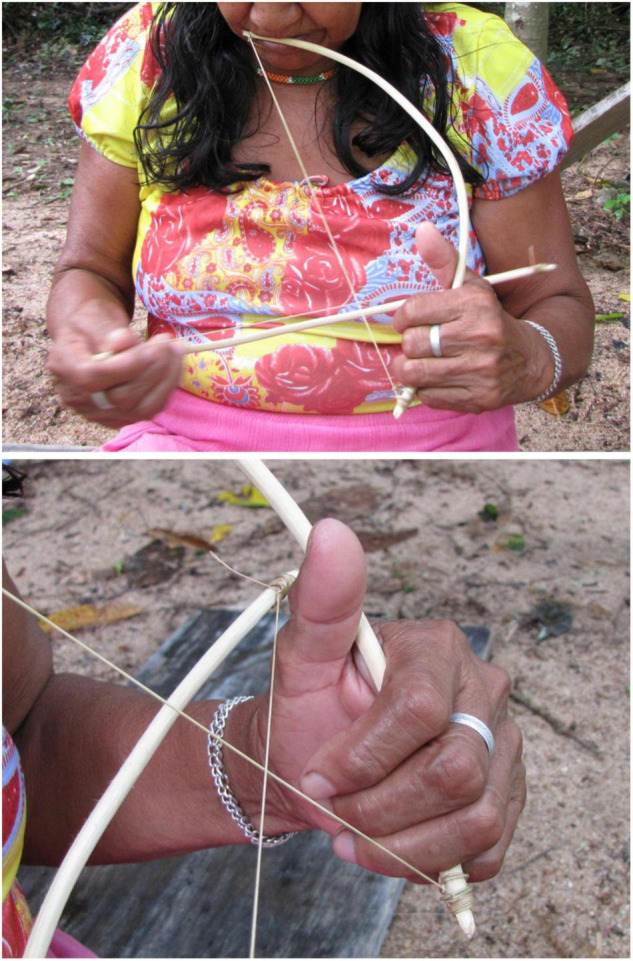
The *iridináp* mouth bows are played exclusively by women. This musical instrument is based on the principle of rubbed strings: the player applies with the fingers three levels of tension to a single cord rubbed by the other bow. The mouth of the player is also used as a resonance chamber (Photos Laure Dentel/Julien Meyer).

**FIGURE 2:**
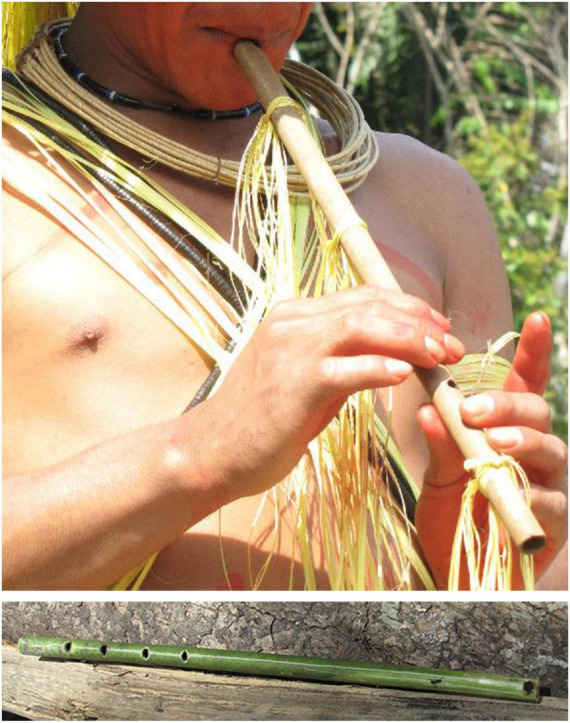
The *kotiráp* flute is an open bamboo stick with five holes: one at one extremity of the instrument in which the players blows and four that are used to produce four different notes (Photos Julien Meyer).

**FIGURE 3:**
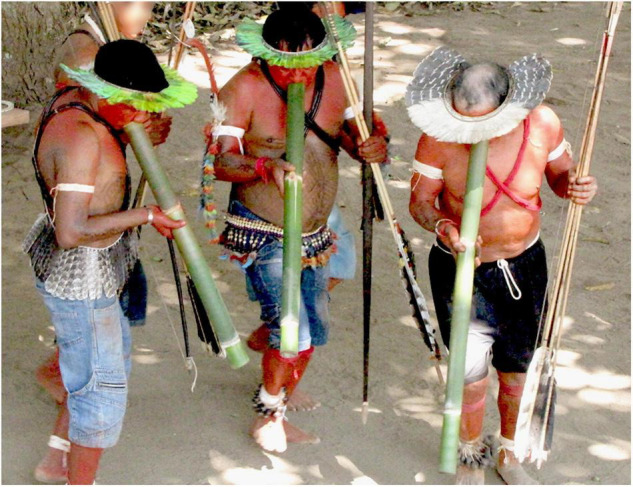
The three bamboo clarinets that constitute the *totoráp* musical instrument are always played together while dancing or sitting. Each player plays a single note clarinet (Photo: Laure Dentel/Julien Meyer).

**FIGURE 4:**
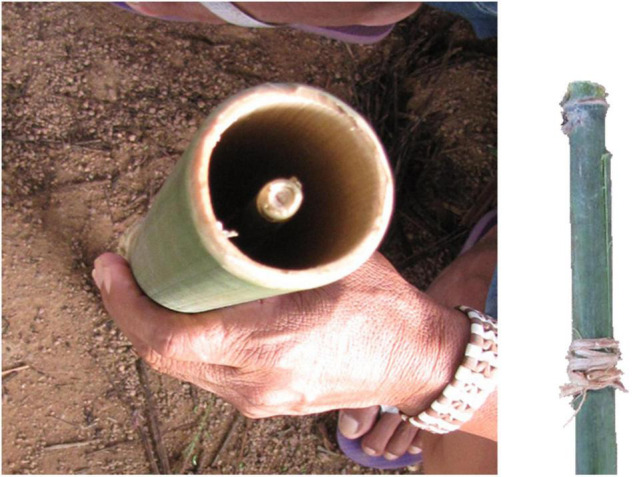
Details of the reed (right), its tuning with a vegetal string and its insertion in the bamboo stick (left) of the *totoráp* clarinets (Photos: Julien Meyer).

During the first step of our inquiry we identified the three different types of instruments that have associated songs. We also verified that there is a large diversity of styles in the musical repertoire of the Gavião: for example, there are also songs that are not played on musical instruments, with a freer relation to linguistic pitch pattern. There is also music that is only played and not sung and that has therefore little to do with linguistic tone. Such music is played on other types of musical instruments (flutes or clarinets with different names in Gavião and different manufacturing processes, for instance). The recording and transcription sessions of music, associated songs and spoken lyrics confirmed the indigenous claim that the *iridináp* musical mouth bows (Figure 1), the *kotiráp* flute (Figure 2), and the *totoráp* clarinets (Figures 3, 4) are expressing the language, and more precisely, the singing mode of speech. All the steps described in the methodology helped identify what the relation is between the music, the song and the spoken pronunciation of the lyrics. The key to this relation is tone and length in Gavião phonology.

We first present here the contexts of use of each instrument and subsequently provide some representative examples of the repertoire of songs that was collected for each type of musical instrument. Next, we extend our pluridisciplinary approach with insights from musical acoustics to characterize the sounds played with each instrument, and with insights from linguistics to understand the music-language relation underlying the musical phrases.

### Ethnographic Enquiry

#### Context of Use and Transmission

Each instrument has its specific contexts of use. For example, the *iridináp* bows are used by the girls and serve exclusively to express messages dealing with love (seduction, refusal and marriage). They are used in private contexts if the boyfriend is near, or during some festivals with all the girls playing and dancing in a line, each one with her instrument. The *kotiráp* flute serves for all types of poetry; it is played by boys and men. It is in general very common for the expression of sentiments of friendship or love or for the description of a distinctive event. It is used in private contexts or in festivals, sometimes to respond to the bows of the girls (Mindlin et al., 2001). Finally, the *totoráp* clarinets are used by boys and men for entertainment in festivals with drink, recounting important events in the life of the community, such as ceremonies, war, poetic impressions or hunting.

Although the children are still learning their maternal language, the instrumental forms of the language are used less and less. The words of these songs frequently refer to traditional knowledge and practices, which now are not well known by a large part of the Gavião population, owing to the reduced frequency of traditional cultural events. Also the Gavião collaborators say that few instrumental songs have been invented since the time of contact, in the decades of 1940–1950. Of the three instruments documented, only the *totoráp* continues with a certain vitality, since it is systematically linked to dances and festivals.

Up till the present, in spite of excellent cooperation with the community owing to confidence in the study of the language, initiated in the decade of the 1970s, only four songs with *iridináp* were found and recorded (with five informants) during the study reported here, eight songs with *kotiráp* (with five informants) and nine songs with *totoráp* (with three groups of three players).

All the informants of the census agree that few songs remain for *iridináp* and *kotiráp*. The eldest at the time of the enquiry (above 70 years old in 2009–2013), who perhaps still knew other songs, had difficulty in passing on the knowledge because they no longer had the force to blow an instrument or the motor coordination necessary to play one. Since much time has passed without playing the instruments of seduction, it is also difficult for the ladies to remember the less popular songs of the past.

#### Repertoire and Some Examples of Songs

The most surprising aspect of the music played by these instruments is that the Gavião spontaneously indicate that it has a meaning. By asking specific questions it is possible to get more details since there is nothing secret about the phenomenon. Generally the players state that the instruments speak words. If one insists, they frequently recount the story told by the lyrics of the music. Other times they sing them. After analysis we realized that the sung version is the key to the instrumental technique because it is exactly that which serves as a model for the music played by the instruments. As mentioned, a linguistic analysis of the words of the sung version shows how they correspond to the notes and rhythms of the played music (see next subsections).

Some examples of the songs are given below. They are short and repeated several times in a musical session. Different players may recombine the components/verses in differing ways, or sometimes delete some parts. Each instrument has its own repertoire of songs. We identified some ancient words in the lyrics and they are in italics in the texts (a), (b), and (c) below. Additionally the songs of *totoráp* and *iridináp* intersperse syllables without meaning (for example, *iriri*, *toy péréré* for the iridináp mouth bows, *s*
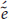
*t s*
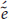
*t, o s*
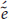
*t o s*
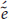
*t* for the totoráp clarinets) for rhythmic instrumental melodies between sung phrases. This characteristic is sometimes found in the songs of the *kotiráp* flute but only with some players (with *o s*
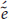
*t o s*
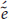
*t*). There are other rhythmic and esthetic processes linked to the tonal melody, which will be explained later in the article.

(a) The *iridináp* musical bows

The *iridináp* song which everyone knows is the following:


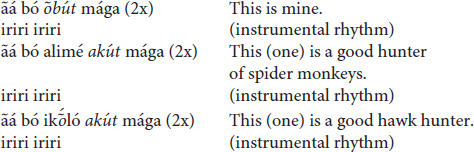


Sometimes the melody is associated with another phrase which is interspersed between two repetitions of the preceding stanza:


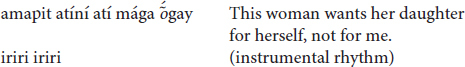


Other songs make subtle reference to the prestige of the hunter, like this one:


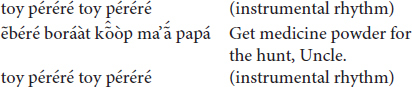


(b) The *kotiráp* flute

The *kotiráp* flute frequently speaks of love and marriage:


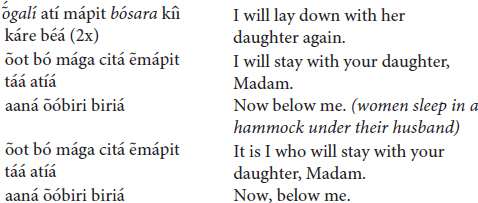


It is also common for the words to refer to a myth (recounting here a summary of the myth of the end of the domestication of the boa constrictor).


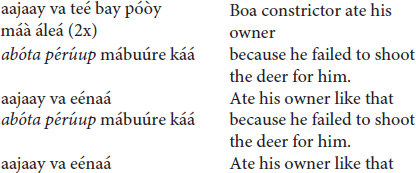


(c) The *totoráp* ensemble of three Clarinets

At this time the *totoráp* song refers to the historical event of the invasion of the Gavião land.


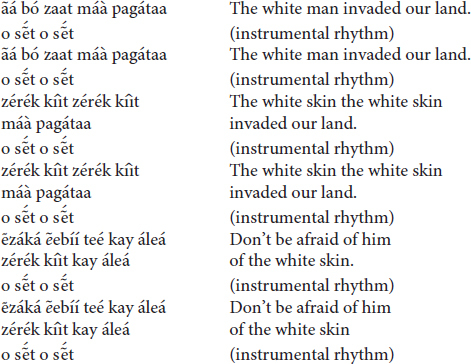


The *totoráp* can also speak poetically to restore a natural environment:


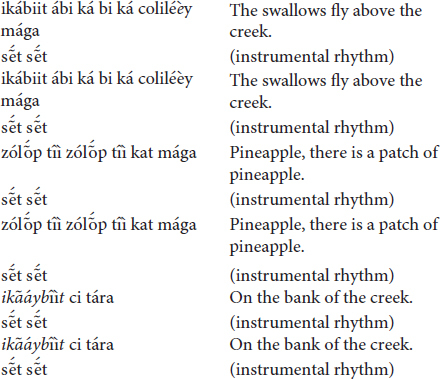


### Musical Acoustics

#### Acoustic Characteristics of the Three Instruments

**FIGURE 5:**
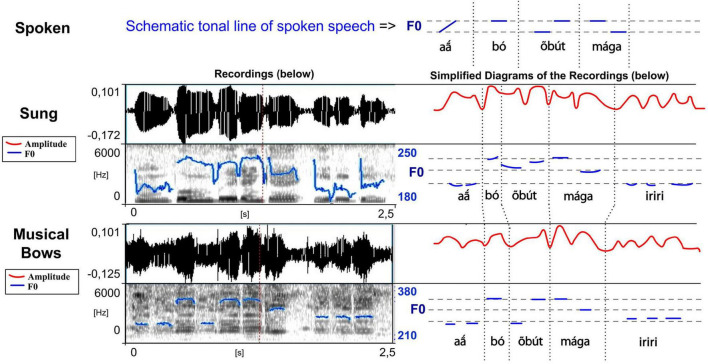
Waveforms and spectrograms of the sung and musical forms of a love song extract played on the Gavião mouth bows (meaning “This [hunter] is mine,” and where *iriri* is just a musical rhythm). The blue lines on the spectrograms represent the extracted F0 (the same parameters have been used for the sung and the instrumental form which explains some jumps in detection in the sung form). For this reason and to see more clearly the similarities between different speech types, they are reported in simplified charts (right), with the amplitude in a red line. The tonal line of the spoken form is also schematically represented (top) (adapted from Moore and Meyer, 2014; listen to sound extracts in Meyer, 2021).

**FIGURE 6:**
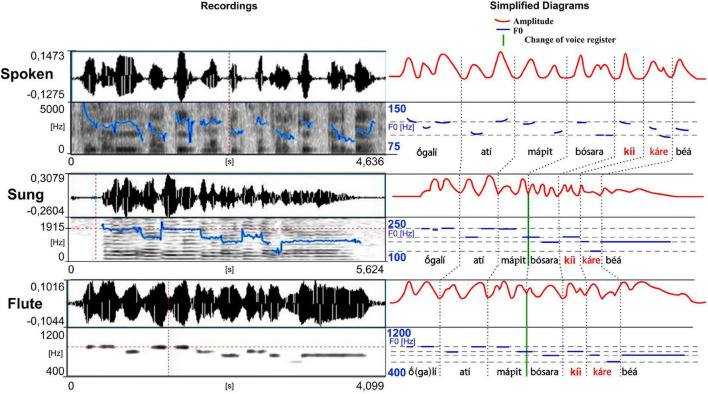
Waveforms and spectrograms of the spoken, sung and musical forms of a song extract played on the *kotiráp* flute (meaning “I will lay down with her daughter again”). The blue lines on the spectrograms represent the extracted F0 (the same parameters have been used for the spoken and the sung speech types which explains more jumps in detection in the spoken form). To see more clearly the similarities between different speech types, they are reported with a focus on vowel nuclei in simplified charts (right), along with the amplitude in a red line. Note the tone downstep after *kíì* in *kíì káre béá*. Note also the change of voice register in the middle of the verse (delimited by a green line) (adapted from Meyer and Moore, 2013; listen to sound extracts in Meyer, 2021).

**FIGURE 7:**
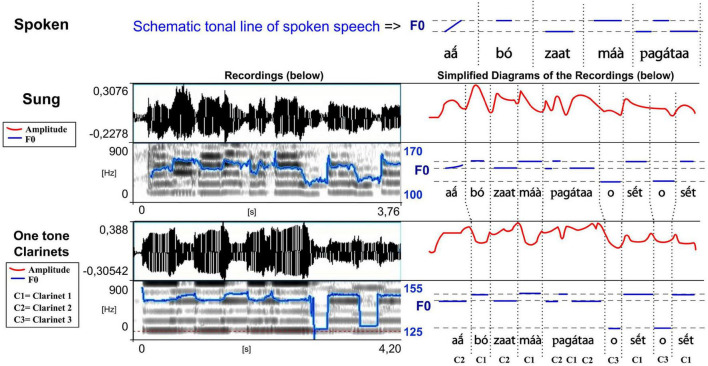
Waveforms and spectrograms of the sung and musical forms of a song extract played on the *totoráp* clarinets (meaning “The white man invaded our land,” and where *o s
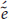
t o s
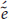
t* is just a musical rhythm). The blue lines on the spectrograms represent the extracted F0 (the same parameters have been used for the sung and musical speech types which explains more jumps in detection in the sung forms). To see more clearly the similarities between different speech types, they are reported in simplified charts with a focus on vowel nuclei (right), along with the amplitude in a red line (adapted from Meyer and Moore, 2013; listen to sound extracts in Meyer, 2021).

The music played with the *iridináp, kotiráp*, or *totoráp* is characterized by the particular timbre of each instrument (Figures 5–7), by the melody of the notes played and by the rhythms of complex musical phrases interspersed with simple rhythms. We won’t provide here an ethno-musicological description, which would be beyond the scope of the present article, but rather an acoustic description of the main points of interest for the music-language comparison. For a direct audio impression of the music of each instrument under study, some audio recordings corresponding to some of the figures illustrating this paper are provided in Meyer (2021), which serves as an audio annex.

First, each instrument has its own acoustic signature, characterized by its timbre. The *iridináp* is an instrument which—like a violin—is based on the principle of rubbed strings: the player applies three levels of tension to a single cord rubbed by the other bow and in this way produces three different musical notes. The mouth of the player is also used as a resonance chamber (Figure 1). The acoustic result is a fundamental frequency of 200–350 Hz and various harmonics equally reinforced (Figure 5). The *kotiráp* is a wind instrument, a flute with 4 holes corresponding to four different notes (Figure 2) with simple timbre, reduced to a sinusoidal signal in the band of 500–900 Hz with its harmonics (Figure 6). The *totoráp* clarinets constitute a wind reed instrument composed of three monotone clarinets (Figures 3, 4), characterized by dense and complex harmonic sounds, with a fundamental frequency in the band of 100–200 Hz with its odd harmonics reinforced (Figure 7). Since the three instruments are harmonic, the height of each note played on any instrument is defined by its fundamental frequency (F0). Note that the perceptual attribute of F0, pitch, constitutes an independent entity parallel to timbre in human perception (Schouten et al., 1962; Risset, 1968; Schwartz and Purves, 2004).

It is through the perception of pitch that the players tune their musical instruments. The manufacturing of these three Gavião instruments is often completely public and a collective work, particularly for the *totoráp*, for which each participant –including the dancers who won’t play the instruments—is temporarily specialized in preparing one of the elements of the three clarinets. All instruments are made with great attention but the manufacturers do not measure them with much precision. In the flute, the four holes are evenly spaced and realized by burning the bamboo with a wooden stick. The two bows of the mouth bow are approximately of the same size and are interchangeable. Concerning each clarinet of the *totoráp* ensemble, they consist of a tube and a reed. The three tubes have generally slightly different sizes. Since we worked in several villages and since our enquiry lasted several years, we had occasion to verify that for all types of instruments (flute, mouth bows and clarinets) a large variation of notes is tolerated. For the mouth bows and the flute, it is because the instruments can change in size and diameter depending of the pieces of wood that are chosen. For the flute, the longer the bamboo, the wider is the space between holes, with the fixed condition that the holes have equal spacing in the lower part of the bamboo. For the clarinets, what is the most important for determining the tonal height is the tuning of the reed, which imposes its frequency to the tube (this is well described for similar instruments among the Wayãpi by Beaudet, 1997). Moreover, differences between different ensembles of clarinets show that three clearly distinct notes are necessary for the instrument to be acceptable but it is not necessary that they are equally distant in frequency or that they keep the same relation from one ensemble to another. Figure 7 shows three stable notes for one *totoráp* ensemble with F0s at respectively 130, 145, and 155 Hz. Other Gavião *totoráp* clarinet ensembles have been documented and show a different absolute distribution of notes each time. For example the clarinet ensemble recorded in the “totorap-AudioSupplement” file available in the audio annex of the present paper (see Meyer, 2021) is characterized by three stable notes at respectively 115, 126, and 134 Hz. This other ensemble is also the one recorded in Moore and Meyer (2014).

In musical acoustics, the form of the variation of amplitude is called “envelope of the wave.” This refers to the graph of the amplitude of the signal, which shows the variation of amplitude as a function of time. The envelope graph of the melodies played by each type of instrument described here is quite similar to the envelope graph of the words of each associated song (and therefore also of their rhythm, which is strongly characterized by the peaks of sound energy), with some differences between the type of musical instruments. The *kotiráp* flute and the *iridináp* double-bow tend to reproduce the outlines of the amplitude graph of the associated song, with the *kotiráp* flute being more precise in this aspect (Figures 5, 6). In the case of the *totoráp*, each of the three clarinets has a level of amplitude that is relatively stable and distinct from the others, which corresponds to the force of the wind applied by the player to vibrate the reed and produce the sound. As explained above, the reeds of the three clarinets are tuned differently: the less the vibrating part of the reed (sometimes adjusted with a vegetal string as shown in Figure 4), the greater the force necessary to make it vibrate and the higher the frequency of the sound which results from the vibration. This constraint in the *totoráp* thus influences and limits the possibilities of emulation of the amplitude envelope of the lyrics.

#### Adaptation of the Sung Mode to the Instruments

**FIGURE 8:**
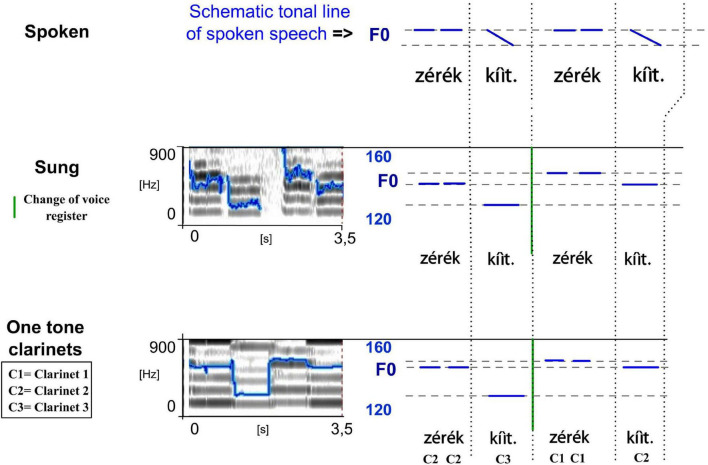
Spectrograms of the sung and musical forms of a song played on the *totoráp* clarinets (meaning “The white skin the white skin.” The blue lines on the spectrograms represent the extracted F0 (the same parameters have been used for the sung and musical speech types which explains more jumps in detection in the sung forms). To see more clearly the similarities between different speech types, they are reported in simplified charts with a focus on vowel nuclei (right). Note here the change of voice register in the middle of the verse (delimited by a green line) and the repetition (adapted from Meyer and Moore, 2013).

**FIGURE 9:**
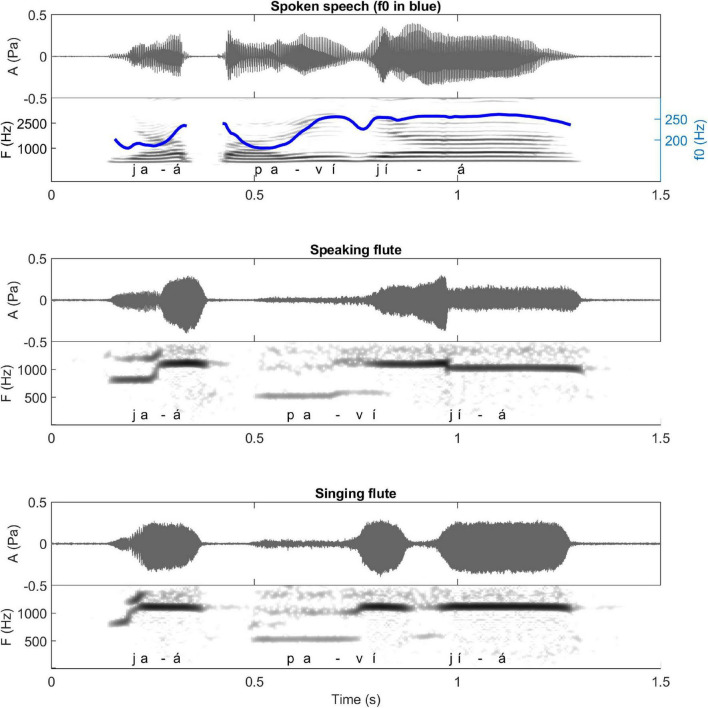
Waveforms and spectrograms of the sentence *jaá pavíjíá* (meaning “Let’s go to bath”) played in spoken speech (upper row), with the flute as if speaking (middle row), and with the flute as in traditional *kotiráp* songs (lower row). The blue line on the spectrograms of spoken speech represents the extracted F0, highlighting the surface tonal line of speech.

The sung forms corresponding to the melodies played with *iridináp, kotiráp* and *totoráp* have particular acoustic characteristics which indicate that they were standardized by the Gavião in order to be played on the instruments. The principle lines of evidence which converge in support of this observation are the following:

In principle, spoken and sung Gavião forms of speech have no limits on the modulation of the fundamental frequency. For example, there are in Gavião culture genera of songs, such as those of the shaman or of festivals, which are not associated to the playing of a musical instrument and which use modulation of F0 both for musical ornamentation and for encoding the tones (Moore and Meyer, 2014). By contrast, the tones of the sung forms of the songs of *iridináp*, *kotiráp* and *totoráp* are realized without internal contours (modulations). For the three bamboo one-tone (one-note) clarinets, the impossibility of producing frequency modulations with such an instrument would explain why the associated songs produce sequences of flat notes (the rising spoken tone of the initial *ãá* of the verse *ãá bó zaat máà pagátaa* is played as flat low note (Figure 7) and the falling spoken tones of the repeated expression *zérék kíìt* are played as flat lower notes than the ones of the preceding word, see Figure 8). The music with flute and mouth bows shows the same absence of modulations, even in the associated sung forms, despite the fact that modulations are technically possible to produce with such instruments, at least micromodulations. Interestingly, collected data shows also that even with the flute, such modulations are not easy to produce. For example, when we asked the players to try to “speak with the flute” the common sentence *jaá pavíjíá* (“shall we go to bathe,” as shown in Figure 9) that does not appear in any *kotiráp* flute traditional song, we observed that instead of producing a long note emulating the long rising tone spoken for *jaá*, they produced two level notes with a micromodulation in between (Figure 9, middle row). By contrast, when asked to produce the same sentence as it would be traditionally sung, they rather produced a high flat note (Figure 9, lower row). It appears that in the latter case relative height is being maintained in relation to the surrounding tones (the *jaá* is higher than the *pa*).

For the flute and the mouth bows, the Gavião players thus deliberately choose to focus instead on the production of level notes for singing the verses. This has important consequences for playing the tone in Gavião language, including adaptations of several tonal rules (see section “Correspondence Between Musical Notes and Phonetic Tone—Playing Flattened Surface Tone”).

A second important aspect in relation to tone levels is that the sung form is adapted to the given number of different notes played on each instrument, as illustrated in Figures 5–7 (three notes for the *totoráp* and *iridináp* instruments and four notes for the *kotiráp*). In the case of the *totoráp*, since each player represents a part of the instrument and executes only one note, this also influences the melody, with the goal of having participation by everyone in the phrase that is played (Figures 7–9). Notably, when a low tone is played on vocables, these low tones are played with the lower note of the instrument, as if it were a kind of style marker of nonsense vocables.

#### Some Specific Esthetic Effects

**FIGURE 10:**
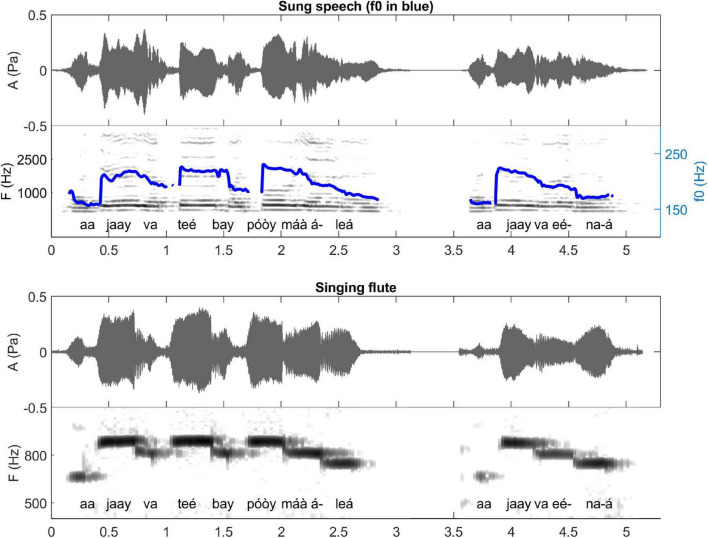
Waveforms and spectrograms of two different verses of the same “Boa constrictor” song traditionally played with the *kotiráp* flute among the Gavião people. The first verse is *aajaay va teé bay póòy máà áleá* (meaning “Boa constrictor ate his owner”), the second verse is *aajaay va eénaá* (meaning “ate his owner like that”). The blue line on the spectrogram of sung speech (upper row) represents the extracted F0 that is emulated by the flute on the lower row. Note that *máà-á* are played together as realized in normal speech, and that *va eé* are also played together, as realized in fast speech (see explanation in section “Correspondence Between Musical Notes and Phonetic Tone—Playing Flattened Surface Tone” listen to sound extracts in Meyer (2021).

First, like all the types of Amazonian songs, the music played by the three analyzed instruments uses various types of repetition:

– repetition of a whole phrase (several examples can be found in the music lyrics above in section “Repertoire and Some Examples of Songs”);
– repetition of one part of a phrase, as in *ikábiit ábi ká bi ká*, but also in *zól
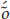
p tîì zól
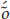
p tîì* (in the second *totoráp* song of section “Repertoire and Some Examples of Songs”) or in *zérék kíìt zérék kíìt* (see the first song of section “Repertoire and Some Examples of Songs” and the illustration in Figure 8).
– repetition of the whole song several times
– the music of these singing instruments also widely uses motif repetitions—a recurring set of notes with the same temporal intervals—which may be the result of different lyrics in the same song. That is to say, words are chosen which will repeat a musical pattern already established by other words (this is well illustrated by the Figure 10, where the three notes of *póòy máà á-leá* follow the same melody and rhythmic patterns as the three notes of *jaay va eé-naá*; see also details on how these examples are played in section “Correspondence Between Musical Notes and Phonetic Tone—Playing Flattened Surface Tone”).

Another common esthetic technique found in these instruments is a sudden lowering or rising of the whole scale of notes used to transpose the tonal levels. Such a technique is akin to the effect of change of vocal register usually found in sung speech, which is characterized by frequency breaks between lower and upper registers [in classical singing, a person’s vocal range is usually thought of in terms of different sections or registers, which arise from different types of vibratory patterns of the vocal chords and thus are categorized in different domains of frequency pitch which may correspond to different pharyngeal shapes and/or different areas of resonance of the voice in the body (e.g., head vs. chest)].

– in the *kotiráp* flute this is manifested as a frequency lowering of one note in the scale of its four musical notes. This lowering happens in the middle of each long verse of music. This can be well observed in the sentence *
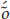
gali atí mápit bósara kíì káre béá* presented as an example in Figure 6. Interestingly here the lowering happens between the low tone of the syllable *pit* and the high tone of the syllable *bó* which are thus at the same level and played in one sole long continuous note;
– in the case of the *totoráp* and *iridináp* music, this esthetic effect may happen between repetitions of the same phrase or of part of a phrase. This is well illustrated for the *totoráp* clarinets in Figure 8 showing a focus on the difference between two repetitions of the expression *zérék kíìt* (here, the second repetition of this expression is higher in pitch). On both repetitions, the long vowel of the syllable *kíìt* is played with the clarinet as a long flat tone that is lower than the ones of the preceding high tone syllables *zérék*—adapting the music to the phonological rules explained in section “Some Phonetic and Phonological Characteristics of the Language of the Gavião of Rondônia” for a floating low tone at the end of an utterance, but also adapting to the constraints of the instrument in terms of modulation as we just explained earlier (see more details in “The Linguistics of the Music-Language Relation” for linguistic details/implications of this adaptation). Note also that the *totoráp* and the *iridináp* music are based on three discrete notes and the musical transposition of a change of voice register is thus more limited than for the *kotiráp* flute.

### The Linguistics of the Music-Language Relation

#### General Considerations, Similarities and Differences Between Spoken and Musical Lines

In speech there is a distinction between fundamental frequency (F0)—which corresponds to the vibration of the vocal cords and carries the supersegmental prosody (including the lexical tone in the case of tone languages)—and its harmonic resonances in the vocal tract, which define the quality of the vowels and of the consonants. We already saw that a distinction between timbre and F0 exists also in the acoustics of the instruments. Based on this parallel, one of the most notable similarities observed between the music played by the *kotiráp*, *totoráp*, and *iridináp* instruments and spoken speech is the syllable-by-syllable correspondence between the respective tones and notes of the two forms.

However, the correspondence is from a spoken tonal line that allows frequency curves (modulations), and an instrumental music line that is based on flat notes (musical constraint). Indeed, the modulations of the melodic line of modal spoken Gavião speech produce curved tones that are hardly ever imitated in the instrumental form, not even in the associated song form (see the topic “Adaptation of the sung mode to the instruments”). This affects the rendering of tonal patterns of the Gavião language by the instruments and is a central part of our analysis.

As we just saw, some other differences observed between the instrumental form and the spoken modal speech form also occur because of esthetic effects that are also present in the sung modality of speech (see the topic of section “Some Specific Esthetic Effects”). Finally, differences come from the use of archaic forms of words in songs which no longer exist in the common spoken form (see the following topic in section “Archaic Forms of Words in the Old Songs”). All of these aspects are emulated by the players of the instruments and coded in the tonal line (F0) of the played melody.

In terms of resemblance, the similarity between normal speech and music is also exhibited in the duration of the spoken syllables and their corresponding notes: the long syllables are made with long sounds on the musical instruments, respecting the quantity distinctions of the Gavião phonology. This is very clear for the *totoráp* clarinets which emphasize precisely this coding level (as shown in Figure 7 for example). For the *kotiráp* flute, relative durations are also respected but with less emphasis than with the *totoráp* clarinets (as shown, for example, in Figure 6 for *kíì* which is one of longest syllables played in this verse). Finally, with the *iridináp* bows a long vowel is sometimes played with two successive notes (see, for example, the two first notes of Figure 5). Moreover, as explained earlier, at the rhythmic level, the dynamic of the amplitude envelope of the instrumental modality of speech parallels the sung one and even the spoken one, with more precision for the flute and less for the clarinets (Figures 5–7).

Finally, the phonetic and phonological nature of the acoustic iconicity which exists between the words of the songs and the music played with the *totoráp*, *kotiráp* and *iridináp* is simply based on the cognitive association between, on one side, the tone and syllable length of the spoken language and, on the other side, the notes played with musical instruments. What could not be foreseen is just how the flat notes reflect the tone and length. As it turns out, there are quite consistent rules for this, and they are common to the three types of singing Gavião instruments.

#### Correspondence Between Musical Notes and Phonetic Tone—Playing Flattened Surface Tone

The variations of F0 of the musical notes correspond directly to the variations of F0 of the lyrics of the associated song (see Figure 5–7), which also follow the variations of F0 of the lexical tone of normal speech. Two major patterns of correspondence are discussed below.

##### Downstep

**FIGURE 11:**
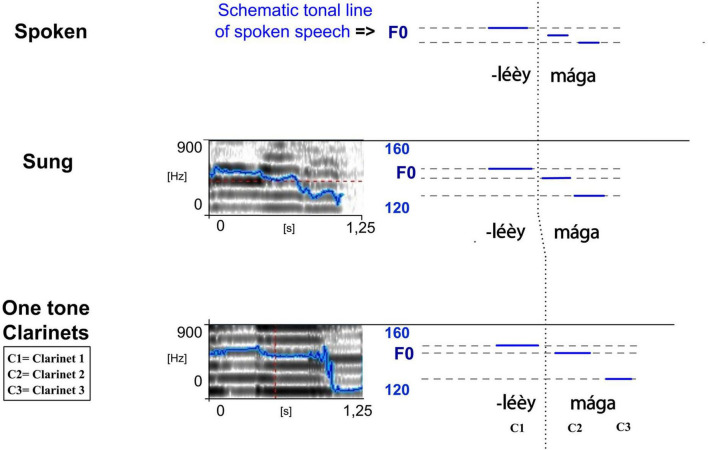
Spectrograms of the sung and musical forms of the song extract *léèy mága* played on the *totoráp* clarinets (from *coliléèy mága* meaning “there are swallows”). The blue lines on the spectrograms represent the extracted F0 (the same parameters have been used for the sung and musical speech types which explains more jumps in detection in the sung forms). To see more clearly the similarities between different speech types, they are reported in simplified charts with a focus on vowel nuclei (right). Note here the tone downstep after *éèy* (adapted from Meyer and Moore, 2013).

The complex phenomenon of the downstep of the Gavião phonology is reproduced by notes which reproduce the effects of this phonological rule. This is exemplified for the *kotiráp* flute in Figure 6 and schematized below in (1) (the downstep occurs after *kíì* in *kíì káre béá*). It is illustrated for the *totoráp* clarinets in Figure 11 and schematized below in (2) (the downstep occurs after *éèy* in *coliléèy mága*). We will see in the discussion (section “Discussion and Conclusion”) that the finding that downstep is preserved in the instrumental speech forms of the Gavião has an important implication: it directly questions previous theoretical hypotheses maintaining that only underlying forms of tonal systems are emulated in speech surrogates (as stated by Bagemihl (1988), who had built such a conclusion on the fact that at the time of his review on the subject he had not found in the literature any mention of surface form imitation in such instrumental systems). Gavião instrumental speech forms are among the rare speech surrogate practices where downstep has been found so far (see other references in the Discussion section 4).

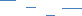

*kíì káre béá (1)* (see also Figure 6)

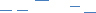

*coliléèy mága (2)* (see also Figure 11)

In the case of (1), the floating low tone of the syllable *kíì* is followed by the high tone of the syllable *ká*, and thus attaches to the following high tone: the combination is realized phonetically as a mid-tone in spoken speech and sung speech, and as a middle note in instrumental speech played with the flute (this is also highlighted in red on Figure 6). This example also shows that the consecutive high tones of the same sentence are also lowered to a phonetic mid-level (here and in Figure 6 see the middle note played to encode *béá*). The case of (2) follows the same pattern and together with the Figure 11 provides a zoom on how these syllables are sung and played with the three clarinets.

##### Rising Tones

Another interesting aspect of the correspondence between musical notes and phonetic tone is that long contour tones are reproduced as flat notes, according to certain rules which take into account the immediate tonal environment, maintaining approximate relative heights. Phonetic long rising tones, whether from fused LH sequences, constant long rising tones, dissimilating long low tones or rising downstep tones, are played as long middle notes when following high or rising phonetic tones and preceding low phonetic tones, as middle or high notes between low phonetic tones and as long low notes finally. These patterns also apply to phonetic curved tones formed by fusing syllables in rapid speech that are separate in slow speech.

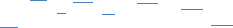

*aajaay va teé bay póòy máà áleá (3)* (see also Figure 10)

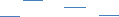

*aajaay va eénaá (4)* (see also Figure 10)

In example (3) and Figure 10 the second syllable of *aajaay* first becomes a phonetic long rising tone because of the low dissimilation rule and then, being a long rising tone between two low tones, is played as a long high note. The long rising tone of *teé* also is played as a long high note between two low tones (note that with the flute the low tones of *va* and *bay* are played as middle notes, on an absolute scale, keeping the relative relation between tones in their environment, while the initial long low and final long rising tones of the sentence are played as long low notes on an absolute scale). The “future” particle *ále* cliticizes to the (downsepped) auxiliary *máà*, producing a descending tone sequence which is played as a long middle note. The final rising tone of *áleá* is played as a long low note.

In example (4) the form *eénaá* cliticizes to the verb *va* and in the resulting form, *veénaá*, the phonetic long rising tone is played as a long middle note followed by a final long low note.

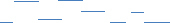

*ẽzáká ẽebíí teé kay áleá (5)*

In (5) the two short high tones of *ẽzáká* are played together as a long high note. The long rising tone of *teé* is played as a long middle note after a high and before a low. That level carries over to the first note of *áleá*. (listen to audio “totorap-Equation5” in audio annex Meyer (2021).

#### Archaic Forms of Words in the Old Songs

As noted earlier, the lyrics of the associated songs contained archaic forms, which require additional explanations. It is interesting to observe what the differences are between the archaic forms (marked in italics in the texts in “Repertoire and some examples of songs”) and the modern forms. In the phrase presented in Figure 4, for example, there appears the verb *bósara*, translated as “going to bed (retiring to a hammock),” which is a lexical item that does not exist in the modern Gavião language. Unexpectedly, the phrase in Figure 6 contains the form *õbút* “1s-thing + diminutive,” which retains the prefix õ-. In the evolution of the languages of the Mondé branch, this prefix disappeared some time ago in the speech of the Gavião and Zoró while persisting in the language of the Paiter (Suruí) and, optionally, in the speech of the Aruá. So, one of the differences in the common archaic forms is in the lexicon and another is in the morphological processes. Since contemporary music composed by the Gavião does not contain these archaic forms they may indicate that the composition of the associated songs occurred centuries ago. However the Gavião can pronounce these old lexical items in normal speech and they conform to modern Gavião phonological patterns.

## Discussion and Conclusion

The Gavião singing instruments, aside from constituting an important cultural heritage threatened by extinction, represent a phenomenon in the interface between language and music. In Amazonia this type of practice is still little known by linguists and musicologists because it is on the margins of each of the two disciplines. In the present article we show the principal steps of our methodology for documenting and studying it. The resulting archives, the associated publications and the collaborative work with the local community represent a contribution to the cultural and linguistic memory of this group, important for revitalization. The Gavião know that the songs played with musical instruments have the rare quality of imitating the sung voice and have a certain pride in their capacity to imitate linguistic properties with musical sounds.

The analytic perspectives are multiple and pluri-disciplinary. We show that techniques of linguistic analysis make it possible to explain, in detail, the nature of the music-language relation in the *totoráp, iridináp* and *kotiráp*, in which lexical tone and syllable length constitute fundamental traits. We highlighted that this relation is due to the fact that linguistic tone is important in a tone language for the understanding of the meaning of the lyrics of the songs (much more than in a language without tone). Very strikingly, because spoken surface tonal downstep (though not the underlying tones with their floating lows) is systematically transposed in this kind of Gavião music, our description also places Gavião instrumental speech practices in a noteworthy situation in the typology of instrumental language surrogates associated with a tone language. Indeed, according to the few existing surveys of the literature about instrumental speech and language surrogates with musical instruments (Sebeok and Umiker-Sebeok, 1976; Bagemihl, 1988), the phenomenon of downstep was not found until recently in instrumental speech forms emulating a tone language. Up to now, as far as we know, it was described first in Serepewa Luth playing Akan language (Nketia, 1994), in Gavião instrumental speech forms (Meyer and Moore, 2013) and, more recently, in Yoruba talking drums (Akinbo, 2020), as well as in Northern Toussain Balafon (Struthers-Young, 2021) and sometimes in singing Balafon forms of Seenku language (McPherson and James, in press). Some recent results analyzing the statistical correspondence between tones of Mooré language and the way they are beaten with the traditional royal Bendré drum also suggests congruence with the surface terracing properties of the tonal Mooré system (Dentel and Meyer, 2020) but confirmation with detailed linguistic analysis remains to be done.

It does appear that the great majority of speech surrogates diverge from the surface realization of a base utterance and instead encode the underlying tonal phonology of the language that is emulated with the instruments [as reviewed by Bagemihl (1988) and described for example in Ewondo Drumming (Neeley, 1999), or more recently in Sambla Balafon emulating the spoken form of Seenku language (McPherson, 2018)]. So it is something of a rarity that downstep is played in instrumental speech music. Yoruba talking drums were, for example, described as permitting the drummer to regulate gradient pitch, following the contours of Yoruba post-lexical tone. Such versatility in production may explain the behavior observed on downstep with this speech surrogate. Notably, the talking drums have the particularity of enabling diverse frequency modulation patterns because of the ability of the drummer to change the tension of the skin by pressing chords with his arm.

The explanation for Gavião instrumental speech forms is necessarily different. The Gavião downstep is also post-lexical, but it is even emulated on instruments such as the ensemble of three one-tone clarinets that do not have the capacity for any modulation in frequency. Its realization is consistent with a strong generalization about Gavião song emulation: the tone of the surface phonetic form is replicated, but only with flat notes, which reflect the relative levels of the syllables. This constraint affects all contour tones, rising and falling. If the underlying H:(L) sequences of high downstep tones were reproduced, for example, a HL contour within one syllable would be produced, violating the constraint. However, a surface drop to flat mid-level on the following syllable is acceptable and can be played. Interestingly, even a late rule like low tone dissimilation can occur in lyrics to produce a surface rising tone that is then flattened in its instrumental replication, like any other phonetic rising tone. More surprising is that the results of fast speech fusions, which are far from the underlying forms, can be played as flat notes according to the same patterns.

The example of Gavião suggests that the instrumental realization of downstep may depend, across languages, on general constraints on instrumental song representation in the particular language and also on what kinds of underlying and surface forms are involved. In the Gavião words the downstep-triggering syllables have underlying floating lows, which only surface as lows in isolated words or elsewhere when they are utterance final (as in the final L tone in the spoken form of the second word in *zérék kíìt*), not medially in connected text. Interestingly, all these cases confirm the need for a reexamination of the theoretical basis for the hypothesis that tonal downstep (as well as downdrift) is absent in instrumental speech surrogates (see Bagemihl, 1988).

In the context of a current effort to compare such “talking/singing” musical practices worldwide, the Gavião language clearly provides new key elements about which levels of the phonological/phonetic structure are possibly accessed and highlighted by talking musical instruments. In that sense, the analysis of the Gavião “singing” instruments contributes to general theory about the principles of instrumental imitation of speech (e.g., McPherson, 2019) and more generally to the typology of tone-tune association in songs based on tonal languages (e.g., Wong and Diehl, 2002; Meyer, 2007; Schellenberg, 2012; McPherson and Ryan, 2018; Ladd and Kirby, 2019). More precisely, by describing new patterns, it adds empirical data to understand better which levels of the phonological/phonetic structure may be transposed in instrumental speech, and how parts of these levels may be represented.

Another point of interest raised by the sounds associated to the music analyzed in this study deals with the syllables without meaning interspersed in the songs. These are akin to vocables because the singers affirm that they represent the sounds and the rhythm played on the instrument itself. They were found for the *iridináp* and the *totoráp* instruments and in each case under two different forms. The vocables corresponding to *iridináp* mouth bows were *iriri* (with first syllables in common with the name of the instrument) and *toy péréré*, while for the *totoráp* ensemble of clarinets they were *s
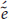
t s
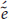
t* and *o s
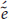
t o s
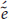
t*. This is a very limited sample that does not enable us to draw definitive conclusions about how and why the vowels and consonants are chosen for each kind of vocable.

When comparing these data with results presented in other studies dealing with larger collections of vocables in traditional song around the world (e.g., Hinton, 1980, 1984; Hughes, 2000; Tuttle, 2012), we did not find a tendency indicating a possible convergence in the acoustic iconicity of these Gavião vocables with common hypotheses of either intrinsic pitch or intrinsic F0 (stating for example that the Formant 2 (F2) or the fundamental frequency (F0) of vowels may influence the choice of the vowel type for such vocables). There are other Gavião musical instruments which also employ vocables and the study should be extended to these in order to have a clearer view on this topic.

Another important factor in the music-language iconic relation is the fact that the songs associated with the instrumental melodies of *totoráp, iridináp*, and *kotiráp* adapt themselves to the particular acoustic properties and modes of playing the instruments (the number of possible discrete notes and/or the lesser possibility of modulating the frequency of the notes), and to certain requirements in the manner of playing them (as in the case of the *totoráp*, which needs the coordination and participation of three people). This absence of frequency modulations in Gavião instrumental singing appears to be a deliberate choice for the flute and the musical bows because it is technically possible to produce at least some elements of modulations with these two instruments, though with some difficulty. We could think of many different reasons to explain that musical behavior: the influence of the *totoráp*, a technical choice to simplify the corpus for non-expert players, a compositional rule for all “singing” instruments or a result of an ancient evolution of singing styles. The closest dialect to the speech of the Gavião, that of the Zoró, as well as the sister language of both, Paiter (Suruí), have no rising tones.

Aside from the average age of the few skilled representative players, linguistic facts also converge to demonstrate the antiquity of the phenomenon. There are archaic forms in the Gavião words in the lyrics of the songs associated with the played music, principally in the vocabulary and in the morphological processes. Interestingly, we did not observe any indication of diachronic change in the phonology of the lyrics of the analyzed songs. On the basis of these observations we consider two hypotheses about the songs associated with instrumental speech. First, it is possible that the songs with archaic forms were composed two or more centuries in the past and have maintained their original form. Second, it is also possible that there exists a poetic system of musical composition characteristic of these instruments, capable of producing archaic forms in contemporary times, that is known by the trained players. The next step will be to investigate the possible existence of archaic forms in music of other genera, of recent composition. To clarify these points it is necessary to continue the documentation and the collaboration with the Gavião and other peoples of the Mondé branch of the Tupi family (Cinta Larga, Zoró, Aruá, Paiter) to extend the repertoire of played songs and compare the phenomena typologically.

## Data Availability Statement

The datasets presented in this study can be found in online repositories. The names of the repository/repositories and accession number(s) can be found at: https://soundcloud.com/user-28976943/sets/sounds-ofa-flute-musical-bows-and-bamboo-clarinets-that-speak-in-the-amazon-rainforest.

## Ethics Statement

There was at the time of our research no standing Ethics Committee at the Museu Goeldi, our host institution in Belém. Ethical questions, should they arise, were addressed by a “sindicância” (an internal investigative committee). In Brazilian law and practice, the participating indigenous community indicates, either orally or in writing, their informed consent to the proposed research to the local office of the National Indian Foundation (FUNAI), which in turn transmits that consent, in the form of a document, to the central FUNAI office in the national capital. This office issues written research permits. Our research followed these established procedures. Native local authorities authorized our work in all of the visited communities. Permits were obtained from the National Indian Foundation (FUNAI) and the National Research Council (CNPq). Thus, research was conducted in accordance with the Declaration of Helsinki. Copies of the recordings and the research results were given to the community. Written informed consent for participation was not required for this study in accordance with the national legislation and the institutional requirements.

## Author Contributions

JM proposed the study, did the fieldwork inquiry, collected and recorded the musical pieces and their associated sung and spoken forms, and prepared the figures. DM provided the contacts for authorization of the study and corrected the transcriptions. JM and DM did the manuscriptwork for the working permit in the indigenous territory, transcribed the texts with local Gavião collaborators, analyzed the data, and wrote the manuscript.

## Conflict of Interest

The authors declare that the research was conducted in the absence of any commercial or financial relationships that could be construed as a potential conflict of interest.

## Publisher’s Note

All claims expressed in this article are solely those of the authors and do not necessarily represent those of their affiliated organizations, or those of the publisher, the editors and the reviewers. Any product that may be evaluated in this article, or claim that may be made by its manufacturer, is not guaranteed or endorsed by the publisher.
